# Evaluation of Regional Myocardial Function by Strain and Strain Rate before and after Surgical Repair of Congenital Heart Anomalies

**Published:** 2018-01

**Authors:** Sima Rafieyian, Shahla Roodpeyma, Kourosh Vahidshahi, Alireza Moghadasi

**Affiliations:** *Shahid Modarres Hospital, Shahid Beheshti University of Medical Sciences, Tehran, Iran.*

**Keywords:** *Cardiac surgical procedures*, *Echocardiography, Doppler*, *Elasticity imaging techniques*

## Abstract

**Background:** Tissue Doppler imaging yields useful information about regional myocardial function. The purpose of this study was to investigate myocardial function by strain and strain rate in a group of patients with congenital heart disease (CHD) before and after cardiac surgery.

**Methods: **Three consecutive tissue Doppler echocardiographic examinations were performed on 25 patients with CHD, who underwent open-heart surgery. The study was conducted from April 2013 to April 2014 in a university hospital, and the assessments were done 1 day before and 1 week and 1 month after surgery. The effects of demographic variables, types of anomalies, and cardiopulmonary bypass factors on strain were evaluated.

**Results:** The study population comprised 13 female and 12 male patients at a mean age of 9.4 ± 9.8 years. Compared to the preoperative data, repeated measurements of strain in 9 segments of the ventricles showed a significant reduction 1 week after surgery, followed by a significant augmentation 1 month postoperatively (p value = 0.001 for all 9 segments). The reduction in strain at the middle segment of the left ventricular free wall was significant in the cyanotic patients (p value = 0.037). The increase in strain at the middle segment of the septum and the right ventricular basal and middle segments was significant (p value = 0.021, p value = 0.015, and p value = 0.021, respectively) in the patients with a shorter pump time.

**Conclusion:** Our patients experienced an early decline in myocardial function after cardiac surgery, but their myocardium recovered its contractility gradually.

## Introduction

Strain and strain rate echocardiography represents a new modality by comparison with noninvasive cardiac imaging methods based on tissue Doppler ultrasound. Considering the physiology of the cardiac muscle, strain is directly related to fiber shortening and strain rate to the velocity of shortening, which is a measure of contractility. The measurement of myocardial strain and strain rate by tissue Doppler imaging (TDI) yields useful information about regional myocardial function, and the assessment of strain and strain rate by TDI is, in fact, imaging wall deformation. It is an objective and measurable technique for the evaluation of systolic and diastolic functions as well as global and regional ventricular functions.^1^^, ^^2^ Similar to left ventricular (LV) ejection fraction, which quantifies global contractility, myocardial strain quantifies regional contractility. Strain and strain rates are, however, essentially similar between apex and base. Strain and strain rate in principle are superior to velocity as the markers of regional contractility. With the present state of technology, velocity imaging is preferred as the primary TDI methodology for assessing regional function. Strain and strain rate imaging may be used as a supplementary method for exploring changes in regional function. Myocardial velocity and strain imaging can be applied clinically to diagnose myocardial ischemia; furthermore, they can be used for the quantification of ventricular dyssynchrony.^3^ Strain and strain rate measurements can be performed at different points within the myocardium; they offer the advantage of allowing the assessment of intrinsic local myocardial function. Tissue Doppler-derived longitudinal strain rate correlates with the extent of myocardial fibrosis. Strain rate imaging seems feasible and effective for the assessment of myocardial viability.

In the present study, regional myocardial function was analyzed using strain and strain rate echocardiography. Regional myocardial function was assessed in a group of pediatric and adult patients with congenital heart disease (CHD), who underwent cardiac surgery. The aim was to compare myocardial deformation before and after cardiac surgery and also to evaluate the effects of demographic variables, types of cardiac anomalies, and cardiopulmonary bypass factors on myocardial strain.

## Methods

This was an observational study (before and after cardiac surgery) by prospective data collection. The study population consisted of pediatric and adult patients with CHD, who underwent open- heart surgery at Shahid Modarres Hospital (a tertiary referral university center) in Tehran, between April 2013 and April 2014. During the 1-year period of the study, 150 open-heart surgical operations were performed. Patients with complete bundle branch block, those with severe cardiovascular events such as cardiac arrest and resuscitation at the operating room or at the intensive care unit, and the ones who were lost to follow-up at appointed times were excluded from the study. Only patients with complete medical records who adhered to the appointed schedule for regular follow-ups were included in this study. During the study period, 25 consecutive patients with the above criteria were enrolled. All the patients underwent complete physical examinations, chest X-ray, electrocardiography (ECG), and echocardiography. All the pre- and postoperative echocardiograms were obtained by a single experienced echocardiologist using a Vivid 7 scanner (Vingmed, General Electric, USA). The preoperative echocardiograms were obtained in all the patients 1 day before surgery and the postoperative echocardiograms were performed 1 week and subsequently 1 month after surgery. One week after surgery, all the patients were in stable condition without mechanical ventilation or inotropic support. All the patients were discharged from the hospital at the time of the 2nd postoperative study (1 month after surgery). First, the conventional echocardiographic examinations including M-mode, color, and pulsed-wave Doppler were performed. Next, color Doppler myocardial imaging was obtained in the standard apical 4-chamber view in all the subjects. The sector size and depth were chosen to achieve the narrowest possible ultrasound window and the highest frame rate (130 ± 20 frames/s). The rate of angulation between myocardial motion and the ultrasound beam was kept at less than 20^°^. The right ventricular (RV) and LV free walls and the interventricular septum were separately imaged from an apical view to assess the longitudinal wall excursion. The sample volume was placed in the basal, mid, and apical segments. Peak systolic longitudinal strain (percentage in the segment length from end diastole) and peak systolic longitudinal strain rate (representing the rate of deformation) were recorded for each segment. Cardiac cycles were recorded during normal quiet respiration. Three consecutive cardiac cycles were recorded and stored digitally for offline analysis. Also evaluated were the effects of demographic variables including gender, age (< 5 y and ≥ 5 y), types of CHD (acyanotic and cyanotic), cardiopulmonary bypass time (pump time < 90 min and ≥ 90 min), and aortic cross-clamp time (< 60 min and ≥ 60 min) on regional deformation of the myocardium at 1 week and 1 month after surgery. Before enrollment in the study, sufficient information about the type of surgery and the importance of scheduled follow-ups were explained to the study population. Informed consent was obtained from the patients or their parents. The Medical Ethics Committee of Shahid Modarres Hospital approved the study.

The data analyses were performed using SPSS, version 22.0 for Windows (SPSS, Inc. Chicago. IL, IBM). All the values are presented as means ± standard deviations. Repeated measurement analysis (Mauchly’s test of sphericity, sphericity assumed, and Greenhouse–Geisser) and Wilcoxon tests were used to compare the preoperative data with the postoperative data. Comparison between the independent groups was performed using the Mann–Whitney test. A p value < 0.05 was considered the criterion for statistical significance. 

## Results

The study population comprised 13 (52%) female and 12 (48%) male patients at a mean age of 9.4 ± 9.8 years (range = 1–32 y). The median age was 5 years; 13 cases were younger than 5 years and 12 were older than 5 years. Acyanotic heart defects were found in 18 (72%) patients and cyanotic lesions in 7 (28%). The types of CHD were as follows: 6 cases of ventricular septal defects, 5 cases of atrial septal defects (ASDs), 4 cases of tetralogy of Fallot (TF), 4 cases of aortic valve replacement, 3 cases of pulmonary valve replacement after total correction of TF, 2 cases of atrioventricular septal defects, and 1 case of coarctation of the aorta with subvalvular aortic stenosis. The pump time and aortic cross-clamp time were 91.28 ± 14.4 (62–120) minutes and 61.04 ± 14.4 (32–108) minutes, respectively. The ejection fractions were 45% to 60% with a mean of 53.1%.

The repeated measurements of strain and strain rate parameters in 3 segments (basal, middle, and apical) of the LV free wall, right ventricle (RV) free wall, and interventricular septum at 3 time points (i.e., preoperative and 1 week and 1 month postoperative) showed a statistically significant trend. In other words, there was a reduction 1 week after surgery compared to the preoperative data, followed by an increase in the values 1 month after surgery in all 9 points (p value = 0.001 for all 9 points). Table 1 summarizes the comparison between the preoperative parameters and the 2 postoperative measurements. 

Our data showed that 1 week after surgery, the regional deformations of both ventricles were significantly reduced. We evaluated the effects of the demographic factors, types of cardiac lesions, and cardiopulmonary bypass factors on this reduction. Table 2 summarizes the results. A comparison between the cyanotic and acyanotic patients demonstrated that the reduction in regional deformation in the cyanotic cases was more pronounced than that in the acyanotic ones. Nevertheless, this difference was not significant, except for the middle segment of the LV free wall, where the reduction in strain was significant (-0.27 ± 0.12 vs. - 0.10 ± 0.29; p value = 0.037). Furthermore, in nearly all the points, the decrease in myocardial strain was more severe in the patients with a more prolonged pump time, but this decrease failed to constitute statistical significance. No significant relationship was found between the reduction in strain and the demographic variables (gender and age) and aortic cross-clamp time.

Table 3 summarizes the relationship between the demographic characteristics, cardiopulmonary bypass factors, and increase in strain 1 month after surgery. As can be seen, the increase in strain at the middle segment of the septum and the RV basal and middle segments was significant (p value = 0.021, p value = 0.015, and p value = 0.021, correspondingly) in the patients with a shorter pump time. No significant relationship was found between the increase in strain and age and aortic cross-clamp time.

**Table 1:** Repeated measurement analysis of myocardial strain before and after surgery

Strain (s^-1^)	Segment	Before Surgery	One Week after Surgery	One Month after Surgery	P Value
Left ventricular free wall					
	Basal	-1.39±0.49	-1.17±0.46	-1.40±0.56	0.001
	Middle	-1.36±0.52	-1.20±0.40	-1.44±0.50	0.001
	Apical	-1.39±0.49	-1.17±0.40	-1.45±0.44	0.001
Interventricular septum					
	Basal	-1.41±0.46	-1.22±0.38	-1.48±0.43	0.001
	Middle	-1.39±0.45	-1.21±0.38	-1.47±0.44	0.001
	Apical	-1.38±0.45	-1.18±0.39	-1.46±0.45	0.001
Right ventricular free wall					
	Basal	-1.42±0.49	-1.22±0.40	-1.46±0.45	0.001
	Middle	-1.42±0.46	-1.20±0.38	-1.43±0.51	0.001
	Apical	-1.36±0.51	-1.22±0.39	-1.48±0.44	0.001

**Table 2:** Relationship between the demographic characteristics, cardiopulmonary bypass factors, cyanosis, and changes in myocardial strain 1 week after surgery

	Segment	Left Ventricular Free Wall			Interventricular Septum			Right Ventricular Free Wall		
		< 5 y	≥ 5 y	P value	< 5 y	≥ 5 y	P value	< 5 y	≥ 5 y	P value
Age	Basal	-0.25±0.14	-0.20±0.14	0.470	-0.21±0.24	-0.16±0.15	0.470	-0.19±0.20	-0.20±0.18	0.689
	Middle	-0.14±0.34	-0.17±0.14	0.503	-0.20±0.19	-0.16±0.12	0.376	-0.21±0.16	-0.24±0.14	0.979
	Apical	-0.20±0.23	-0.22±0.12	0.894	-0.21±0.21	-0.18±0.11	0.474	-0.08±0.29	-0.24±0.14	0.979
		Male	Female	P value	Male	Female	P value	Male	Female	P value
Gender	Basal	-0.25±0.15	-0.21±0.13	0.650	-0.24±0.20	-0.14±0.19	0.225	-0.22±0.14	-0.17±0.22	0.538
	Middle	-0.15±0.31	-0.16±0.21	0.810	-0.24±0.14	-0.12±0.17	0.098	-0.24±0.11	-0.20±0.18	0.538
	Apical	-0.24±0.16	-0.18±0.21	0.728	-0.24±0.11	-0.15±0.20	0.376	-0.09±0.28	-0.17±0.17	0.689
		Cyanotic	Acyanotic	P value	Cyanotic	Acyanotic	P value	Cyanotic	Acyanotic	P value
Types of CHD	Basal	-0.24±0.11	-0.22±0.15	0.475	-0.22±0.13	-0.17±0.22	0.475	-0.23±0.12	-0.18±0.21	0.440
	Middle	-0.27±0.12	-0.10±0.29	0.037	-0.23±0.08	-0.15±0.17	0.215	-0.23±0.10	-0.22±0.17	0.754
	Apical	-0.30±0.15	-0.17±0.19	0.110	-0.25±0.08	-0.17±0.19	0.238	-0.16±0.06	-0.11±0.28	0.669
		< 90 min	≥ 90 min	P value	< 90 min	≥ 90 min	P value	< 90 min	≥ 90 min	P value
Pump time	Basal	-0.15±0.11	-0.30±0.13	0.060	-0.18±0.14	-0.19±0.25	0.936	-0.14±0.11	-0.25±0.23	0.110
	Middle	-0.15±0.12	-0.16±0.34	0.225	-0.147±0.11	-0.19±0.20	0.650	-0.20±0.09	-0.24±0.19	0.320
	Apical	-0.19±0.12	-0.23±0.23	0.347	-0.19±0.12	-0.20±0.20	0.470	-0.13±0.07	-0.13±0.32	0.186
		< 60 min	≥ 60 min	P value	< 60 min	≥ 60 min	P value	< 60 min	≥ 60 min	P value
Aortic cross-clamp time	Basal	-0.23±0.18	-0.22±0.10	1.000	-0.21±0.23	-0.17±0.17	0.689	-0.20±0.16	-0.19±0.21	0.852
	Middle	-0.24±0.16	-0.08±0.31	0.376	-0.18±0.16	-0.18±0.17	0.810	-0.26±0.11	-0.19±0.18	0.295
	Apical	-0.26±0.18	-0.17±0.19	0.538	-0.22±0.13	-0.17±0.19	0.728	-0.19±0.08	-0.08±0.31	0.295

CHD, Congenital heart disease

**Table 3:** Relationship between the demographic characteristics, cardiopulmonary bypass factors, cyanosis, and changes in myocardial strain 1 month after surgery

	Segment	Left Ventricular Free Wall			Interventricular Septum			Right Ventricular Free Wall		
		< 5 y	≥ 5 y	P value	< 5 y	≥ 5 y	P value	< 5 y	≥ 5 y	P value
Age	Basal	-0.08±0.37	-0.09±0.12	0.186	-0.05±0.16	-0.09±0.07	0.538	-0.04±0.15	-0.04±0.14	0.470
	Middle	-0.07±0.10	-0.09±0.10	0.810	-0.06±0.09	-0.10±0.08	0.347	-0.07±0.10	-0.08±0.08	0.503
	Apical	-0.04±0.09	-0.08±0.12	0.320	-0.07±0.10	-0.08±0.08	0.503	-0.06±0.09	-0.10±0.08	0.347
		Male	Female	P value	Male	Female	P value	Male	Female	P value
Gender	Basal	-0.07±0.40	-0.07±0.10	1.000	-0.07±0.15	-0.07±0.09	0.852	-0.01±0.14	-0.07±0.15	0.247
	Middle	-0.09±0.11	-0.07±0.09	0.689	-0.03±0.08	-0.12±0.08	0.005	-0.05±0.08	-0.10±0.10	0.186
	Apical	-0.08±0.12	-0.04±0.09	0.347	-0.05±0.08	-0.10±0.10	0.186	-0.03±0.08	-0.12±0.08	0.005
		Cyanotic	Acyanotic	P value	Cyanotic	Acyanotic	P value	Cyanotic	Acyanotic	P value
Types of CHD	Basal	-0.05±0.12	-0.02±0.34	1.000	-0.10±0.13	-0.05±0.12	0.288	-0.04±0.10	-0.04±0.16	0.549
	Middle	-0.10±0.07	-0.07±0.11	0.588	-0.08±0.07	-0.08±0.10	0.932	-0.07±0.09	-0.07±0.10	1.000
	Apical	-0.07±0.07	-0.06±0.12	0.711	-0.07±0.09	-0.07±0.10	1.000	-0.08±0.07	-0.08±0.10	0.932
		< 90 min	≥ 90 min	P value	< 90 min	≥ 90 min	P value	< 90 min	≥ 90 min	P value
Pump time	Basal	-0.006±0.33	-0.0007±0.26	0.609	-0.09±0.08	-0.05±0.15	0.403	-0.10±0.16	-0.0007±0.11	0.015
	Middle	-0.05±0.07	-0.10±0.12	0.202	-0.12±0.09	-0.04±0.08	0.021	-0.10±0.06	-0.05±0.11	0.021
	Apical	-0.03±0.08	-0.08±0.12	0.467	-0.10±0.06	-0.05±0.11	0.058	-0.12±0.09	-0.04±0.08	0.058
		< 60 min	≥ 60 min	P value	< 60 min	≥ 60 min	P value	< 60 min	≥ 60 min	P value
Aortic cross-clamp time	Basal	-0.03±0.11	0.02±0.39	0.186	-0.07±0.14	-0.06±0.11	0.810	-0.06±0.13	-0.02±0.15	0.270
	Middle	-0.04±0.0	-0.11±0.09	0.087	-0.10±0.09	-0.06±0.09	0.110	-0.07±0.07	-0.08±0.11	0.110
	Apical	-0.04±0.08	-0.08±0.13	0.376	-0.07±0.07	-0.08±0.11	0.894	-0.10±0.09	-0.06±0.09	0.894

CHD, Congenital heart disease

## Discussion

The results of the present study revealed a significant reduction in strain rate 1 week after surgery. An evaluation of the effects of gender, age, and aortic cross-clamp time on this reduction showed no significant relationship between strain reduction and those factors. The strain reduction in our cyanotic patients was more pronounced than that in our acyanotic subjects, but the difference was not significant except for the middle segment of the LV free wall. The strain reduction in the longer pump time was more than that in the shorter pump time; the difference, however, was not significant. The absence of a significant reduction may have been due to our limited number of patients. Myocardial damage secondary to preoperative hypoxia or cyanosis, cardiopulmonary bypass, intraoperative procedures, size of prosthetic material, hypoperfusion, surgical incision, and postoperative complications (e.g., arrhythmia, prolonged mechanical ventilation, and changes in ventricular compliance) may explain the reasons for the early reduction in strain. Our results showed that the effects of surgical insults were not similar in all the segments of the ventricles. A report on 24 patients with ASDs 2˚, 1 week after surgery, showed that ASD closure via surgery resulted in reduced RV strain and strain rate; nonetheless, after ASD closure with the Amplatzer, there was no reduction in strain and strain rate. In addition, LV parameters did not change before and after ASD closure with either technique. The authors concluded that the above findings might have been the consequence of surgical injury to the heart and suggested that serial follow-up studies be undertaken to clear whether or not the reduction was temporary.^4^ Our study showed that not only was the reduction in strain temporary but also it improved, even better than the preoperative period, 1 month after surgery. Matsuhisa et al.^5^ studied 16 children with multiple ventricular septal defects postoperatively. Postoperative ventricular global and septal functions were significantly reduced in these children, especially when the defects were closed with large prosthetic materials. Thirteen cases in our acyanotic population underwent surgical closure of ASDs, ventricular septal defects, or atrioventricular septal defects and 4 cases with aortic valve stenosis underwent aortic valve replacement surgery. 

There were 7 cases of TF: 4 of them underwent total correction and the other 3 underwent redo surgery for pulmonary valve replacement. RV heart failure, severe pulmonary valve regurgitation, and tricuspid valve regurgitation were confirmed by cardiac magnetic resonance imaging (CMR) in all the redo cases. The complete surgical correction of TF is rather an excellent palliation insofar as it relieves the problem of cyanosis. Be that as it may, it predisposes the patients to subsequent intervention to resolve the surgical sequelae.^6^ Weidemann et al.^7^ found that after cardiac surgery in TF, peak systolic strain and strain rate in the RV free wall were reduced. The rate of reduction in the basal portion of the RV free wall had a direct correlation with the QRS duration and was a good parameter of RV dilatation. These data indicated that during the follow-up period of postoperative TF patients, frequent CMR can be replaced by strain and strain rate assessment. Sadeghpour et al.^8^ studied 70 adult patients late after TF repair and found a significant correlation between the Doppler-derived mean strains of the RV free wall and RV function as measured by CMR. We know that there are interactions between the RV and the LV. A study by Abd El Rahman et al.^9^ showed that 13 (52%) out of their 25 patients, after TF repair, had LV asynchrony. These patients had right bundle branch block and significantly reduced septal systolic strain. The ECGs of our postoperative TF patients showed complete right bundle branch block and significant reductions in septal strain at the middle segment of the LV free wall, which may have been due to LV asynchrony. More studies are needed on the segmental asynchrony of the LV in postoperative TF patients.

Lunze et al.^10^ compared 36 patients after the Fontan operation with a healthy control group and showed a significant drop in the strain and strain rate of ventricular function. However, the regional atrial contractile performance was better preserved after extra-atrial type Fontan surgery. Elsewhere, Pekto et al.^11^ followed up 33 patients with hypoplastic left heart syndrome 21 days after the Norwood operation and found that strain and strain rate were reduced in the patients postoperatively. None of the surgical factors was found to be able to describe this reduction. Rentzsch et al.^12^ studied 24 patients with D-transposition of the great arteries (mean age = 13 - 31 y) after arterial switch surgery and a postoperative period of 16.9 years. The patients were compared with 22 age- and sex-matched healthy individuals. The authors reported that the strain and strain rate parameters showed a highly significant reduction in all the analyzed segments of the RV and the LV. The findings of the above studies suggest that, in complex heart lesions, the reduction in postoperative ventricular strain can persist for several years. We did not have arterial switch, Fontan, or Norwood operations in our study population and the rapid improvement in myocardial strain 1 month after surgery may have been due to the simple nature of the cardiac anomalies in our cases. We did not find a significant correlation between age, gender, aortic cross-clamp time, and postoperative reduction in strain rate in our patients. In a study by Kempney et al.,^13^ longitudinal LV 2D strain was superior to the ejection fraction in predicting myocardial damage or amendment, as well as symptomatic improvement after aortic valve implantation.

In our study, repeated measurement 1 month after surgery showed a significant increase in myocardial strain. A shorter pump time was significantly related with a higher strain in the middle segment of the septum and the middle and basal segments of the RV. The above finding may have been due to a gradual decrease in the suppressive effects of the prolonged pump time on myocardial strain in different segments of the ventricles. In the acyanotic patients, myocardial strain at the 3 segments of the RV was higher than that in the cyanotic subjects, but the difference was not significant. It may show that the deleterious effects of hypoxemia may be more severe on the RV than on the LV, although the difference in our study was not significant. 

The limitation of our study is its small sample size, which may have limited its power to detect significant differences. The reason for this small sample size was the high rate of loss to follow-up after discharge from the hospital and restrictions in the inclusion criteria. Our population was a mixture of different types of CHD and had the advantage of allowing us to compare the effects of cardiopulmonary bypass factors between cyanotic and acyanotic heart lesions.

## Conclusion

In our study population, there was a significant reduction post cardiac surgery in myocardial regional function. This was a temporary phenomenon, and the myocardium regained its contractility even better than that in the preoperative period. Surgical pump time and cyanotic anomalies had significant effects on the regional reduction in myocardial function.

## Notes:

***This paper should be cited as: ***Rafieyian S, Roodpeyma S, Vahidshahi K, Moghadasi A. Evaluation of Regional Myocardial Function by Strain and Strain Rate before and after Surgical Repair of Congenital Heart Anomalies. J Teh Univ Heart Ctr 2018;13(1):18-23.
